# HOPE springs eternal: lack of HIV superinfection in HIV Organ Policy Equity Act kidney transplants

**DOI:** 10.1172/JCI184326

**Published:** 2024-10-15

**Authors:** Christine M. Durand, Andrew D. Redd

**Affiliations:** 1 Department of Medicine, Johns Hopkins University School of Medicine, Baltimore, Maryland, USA.; 2 Division of Intramural Research, National Institute of Allergy and Infectious Diseases, National Institutes of Health, Bethesda, Maryland, USA.; 3 Institute of Infectious Disease and Molecular Medicine, University of Cape Town, Cape Town, South Africa.

## Abstract

Kidney transplantation from donors with HIV to recipients with HIV (HIV D^+^/R^+^) is an emerging practice that has shown substantial clinical benefit. Sustained HIV superinfection, whereby a transplant recipient acquires a new strain of HIV from their organ donor, is a theoretical risk, which might increase chances of viral failure. In this issue of the *JCI*, Travieso, Stadtler, and colleagues present phylogenetic analysis of HIV from kidney tissue, urine, plasma, and cells from 12 HIV D^+^/R^+^ kidney transplants out to five years of follow-up. Early after transplant, donor HIV was transiently detected in five of 12 recipients, primarily from donors with untreated HIV and high-level viremia, consistent with a viral inoculum. Long-term, donor HIV was not detected in any recipients, demonstrating no sustained HIV superinfection. These reassuring data support earlier findings from South Africa and the United States and further confirm the safety of HIV D^+^/R^+^ transplantation.

## The HOPE Act

Solid organ transplantation is considered the optimal therapy for end-stage organ disease, including in people with HIV (PWH). However, a lack of donated organs constitutes a public health crisis, as people die every day waiting for an organ. For PWH, this organ shortage crisis is exacerbated, with data indicating that PWH have higher mortality on organ waitlists (1, 2) and decreased access to organ transplantation (3, 4).

A potential solution to this access issue was pioneered in 2008 by transplant surgeon Elmi Muller, who performed the first deceased donor kidney transplants from donors with HIV to recipients with HIV (HIV D^+^/R^+^) in South Africa (5). In the United States, her results inspired the reversal of a federal ban on the use of organs from donors with HIV (6). These efforts led to passage of the Congressional HIV Organ Policy Equity (HOPE) Act in 2013 (7). In 2016, the first HIV D^+^/R^+^ transplants were performed in the United States (8), and since then, the practice has continued to expand. A growing body of evidence demonstrates good clinical outcomes of HIV D^+^/R^+^ transplantation (9, 10) as well as improved transplant access for a historically marginalized population (11).

One of the theoretical risks of HIV D^+^/R^+^ transplantation is HIV superinfection: the acquisition of a second, distinct strain of HIV in a person with HIV. HIV superinfection has been described with all modes of HIV transmission, including sexual intercourse and injection drug use (12). In the context of HIV D^+^/R^+^ transplantation, if recipients acquire a new strain of HIV from the donor that is resistant to their antiretroviral therapy (ART), loss of HIV suppression and progression of disease could result. Due to this potential risk, as well as others, the HOPE Act mandates that HIV D^+^/R^+^ transplantation be performed under research protocols (13).

## An in-depth virologic analysis

In this issue of the *JCI*, Travieso and colleagues present a longitudinal, in-depth, virologic analysis of 12 cases of HIV D^+^/R^+^ kidney transplantation performed under the HOPE Act (14). All 12 recipients were on ART with undetectable plasma HIV RNA. Among donors, four of 12 were untreated for HIV, including one donor who was hypothesized to have acute HIV, indicated by a positive HIV qualitative nucleic acid test (NAT) and a negative HIV antibody test. The remaining eight donors were on ART: six with undetectable plasma HIV RNA and two with plasma HIV RNA under 500 copies/mL.

Before transplant, donors had blood, kidney biopsies, and urine collected, and recipients had blood, urine (when available), and iliac lymph nodes (two cases) collected. Free virus was isolated from urine, and renal tubular epithelial (RTE) cells were cultured for up to five weeks to obtain virus. After transplant, recipients had blood, urine, and cultured RTE cells collected at multiple early time points (hours to days) to look for donor virus inoculum and at later time points to look for sustained HIV superinfection. Median follow-up time was 2.25 years (ranging from four weeks to five years). Full-length HIV envelope (env) was amplified from biospecimens using single genome sequencing, and neighbor-joining phylogenetic trees were constructed to characterize genetic lineages. A number of notable findings emerged (14).

**Figure 1:**
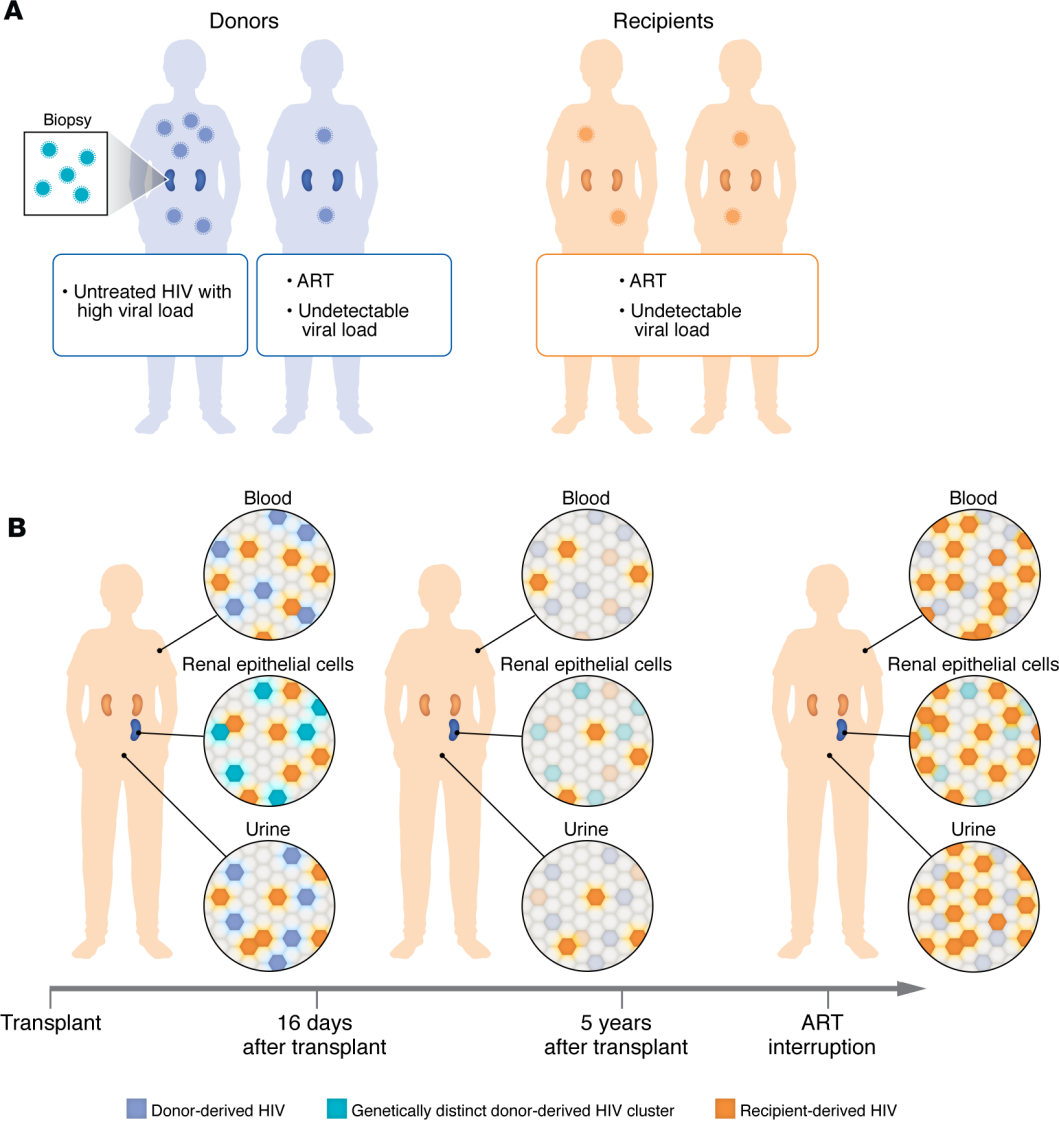
In HIV D^+^/R^+^ transplantation, donor HIV is detected early after transplant, but doesn’t manifest as persistent superinfection. (**A**) Prior to transplant, recipients with HIV are on ART with undetectable plasma HIV RNA levels. In contrast, deceased donors with HIV may or may not be on ART; as such, some will have high-level plasma HIV RNA. In some cases, HIV can be amplified from donor kidney biopsies and occasionally constitutes a separate genetic cluster from HIV in the blood. (**B**) Early after transplant, a mix of recipient and donor-derived HIV can be detected in urine, urine-derived renal epithelial cells, peripheral blood mononuclear cells, and plasma from a subset of recipients. The presence of donor-derived HIV is more common in recipients who receive kidneys from donors with high HIV viral loads and represents a donor viral inoculum. Later after transplant, donor HIV is not detected in recipients, even in those who interrupt ART and experience high levels of plasma HIV RNA rebound. In summary, donor-derived HIV superinfection does not routinely occur in HIV D^+^/R^+^ transplantation, likely due to the protection of ART.

First, Travieso et al. (14) successfully amplified HIV from donor kidney biopsies, in six of 12 cases. These results included four donors on ART with undetectable plasma HIV RNA, one donor on ART with low-level viremia, and one untreated donor with high-level viremia. In three of four cases, where the authors were also able to amplify HIV from donor plasma, they found that HIV from the kidney clustered separately from the plasma (Figure 1A). This finding builds on prior data showing that HIV can be isolated from renal tissue in individuals on effective ART and, in some cases, can form a genetically distinct compartment (15). While interesting, the clinical importance of this result is unclear in the era of viremia-suppressing ART, since there is not clear evidence that the kidney constitutes a long-lived reservoir from which HIV could reactivate (15). Second, in recipients very early after transplant, Travieso et al. detected a mix of recipient and donor HIV in five of 12 recipients, indicative of a transmitted donor inoculum (Figure 1B). The majority of these recipients (four out of five) received kidneys from untreated donors with high-level HIV viremia, with just one recipient whose kidney donor was on ART with undetectable plasma HIV RNA.

Finally, in all recipients, no donor HIV was detected at later time points beyond 16 days (Figure 1B), indicating a lack of sustained HIV superinfection. During follow-up, recipients remained on ART with undetectable HIV plasma HIV RNA, apart from four recipients with viral blips (detectable virus, less than 200 copies/mL). There was a single exception: one recipient who interrupted ART at 3.5 years after transplant and experienced high-level HIV viremia. HIV was amplified during this viremic episode and matched recipient sequences; of note, this rebounding virus was genetically homogeneous, suggesting a mono- or oligoclonal origin, as has been described with typical rebound after treatment interruption (16).

Lack of HIV superinfection and sustained viral suppression aligns with the clinical outcomes data from HIV D^+^/R^+^ transplant studies, where HIV virologic failure has not been reported (9, 10, 17). It also suggests that HIV D^+^/R^+^ transplantation should not substantially increase the size of long-lived HIV reservoirs, which constitute a barrier to HIV cure. This is consistent with studies of the HIV reservoir in HIV D^+^/R^+^ kidney and liver transplantation, which have shown stable reservoir size over time (18, 19).

Prior to Travieso et al. (14), there have been a few in-depth virologic studies on HIV superinfection after HIV D^+^/R^+^ transplantation. In 2019, Selhorst and colleagues presented longitudinal deep-sequencing data on HIV from 25 donor-recipient HIV D^+^/R^+^ recipient pairs, looking both for donor HIV inoculum and persistent HIV superinfection (17). In this study, donor HIV was detected in peripheral blood mononuclear cells from eight of 24 recipients, mostly at one week after transplant, consistent with a donor HIV inoculum. At later time points, donor HIV was only detected in one of 25 recipients; and in this case, the donor virus represented an extreme minority variant (0.002% of sequences) found at week 12, but not isolated later at week 26. The authors reported that this possible superinfection had no clinical relevance for the recipient. In a US study of HIV D^+^/R^+^ kidney and liver transplantation, Bonny et al. also used next-generation sequencing to study this phenomenon (20). This study aimed to quantify the incidence of sustained HIV superinfection and focused on posttransplant time points after week 12. Donor HIV was not detected in any of the 17 HIV D^+^/R^+^ cases out to three years. This study was particularly reassuring, since it also included eight liver transplant recipients (20), which is important since the liver contains the largest population of tissue macrophages in the body and these cells can harbor HIV even in the presence of ART (21). Finally, similarly to the Travieso study (14), that cohort included a liver transplant recipient who stopped ART and had high-level HIV viremia, with only recipient HIV sequences detected at the time of HIV rebound (20). These earlier studies both used next-generation sequencing, which has a higher sensitivity for detection of minor HIV strains than the single-genome sequencing methods used by Travieso et al. However, the fact that Travieso, Stadtler, and authors were able to detect the donor inoculum soon after transplantation, but not at later time points, suggests the sensitivity of the sequencing used here should have been sufficient to detect sustained HIV superinfection.

## Organ transplantation as a mode of viral transmission

Viral transmission in the setting of organ transplantation is distinct from other modes of transmission. With an organ transplant, free virions and provirally infected cells are carried into the recipients from the donor organ tissue and blood. This process exposes the recipient to a higher viral inoculum than traditional modes of viral transmission and has also been associated with other unique virologic consequences. For example, organ transplantation from donors with hepatitis C virus (HCV) to recipients without HCV (HCV D^+^/R^–^) leads to universal acquisition of HCV in the absence of antivirals (22). Moreover, in-depth genetic studies of HCV in this setting have demonstrated that recipients acquire a genetically diverse infection, similar to what is seen in chronic HCV. This type of infection is in stark contrast to the genetically homogenous founder virus population that is seen with acute HCV infection (23, 24).

In contrast to HCV D^+^/R^–^ transplantation, in HIV D^+^/R^+^ transplantation, recipients are universally on ART at the time of exposure to donor HIV. As such, the collective data suggest that the recipient’s ART is protective against acquisition of a second strain of HIV. This paradigm aligns with studies of HIV superinfection in PWH who have ongoing behavioral risk factors for HIV acquisition, where ART has been show to greatly reduce the risk of HIV superinfection (12).

In summary, Travieso, Stadtler, and colleagues convincingly show that for transplant recipients on ART, HIV superinfection is not a routine risk of HIV D^+^/R^+^ kidney transplantation, even when donors have high-level HIV viremia. This study is yet another example of the potency and benefits of effective ART and provides additional optimism for expanding HIV D^+^/R^+^ transplantation to standard clinical practice.

## Version 1 10/15/2024

Electronic publication
